# Minimum required distance for clinically significant measurement of habitual gait speed

**DOI:** 10.1186/s12877-025-06064-8

**Published:** 2025-07-05

**Authors:** Myung Woo Park, Sun Gun Chung, Jaewon Beom, Kyung Su Kim, Joonghee Kim, Chul-Hyun Park, Jinkyu Lee, Keewon Kim

**Affiliations:** 1 Department of Rehabilitation Medicine, Seoul Metropolitan Government-Seoul National University Boramae Medical Center, Seoul, Republic of Korea; 2 Department of Rehabilitation Medicine, Seoul National University College of Medicine, Seoul National University Hospital, 101 Daehak-Ro, Jongno-Gu, Seoul, 03080 Republic of Korea; 3 Department of Rehabilitation Medicine, Seoul National University Hospital, Seoul, Republic of Korea; 4 Department of Rehabilitation Medicine, Seoul National University Bundang Hospital, Seongnam, Republic of Korea; 5 Department of Emergency Medicine, Seoul National University College of Medicine, Seoul, Republic of Korea; 6 Department of Emergency Medicine, Seoul National University Hospital, Seoul, Republic of Korea; 7 Department of Emergency Medicine, Seoul National University Bundang Hospital, Seongnam, Republic of Korea; 8 Department of Physical and Rehabilitation Medicine, Kangbuk Samsung Hospital, Sungkyunkwan University School of Medicine, Seoul, Republic of Korea; 9 Department of Rehabilitative and Assistive Technology, National Rehabilitation Center, National Rehabilitation Research Institute, Seoul, Republic of Korea

**Keywords:** Walking speed, Variability, Pose estimation, Gait analysis, Distance

## Abstract

**Background:**

Gait speed indicates morbidity and life expectancy in older adults, but the minimum walking distance for measurement remains unclear. This study aimed to determine the minimum distance required to measure clinically reliable gait speed using a smartphone camera and pose estimation, and whether the distance is influenced by subject characteristics or measurement methods.

**Methods:**

Twenty-four healthy volunteers (≥ 65 years old) performed a video-recorded 10-m gait test, including acceleration and deceleration. Fourteen body points were derived using a pose-estimation algorithm. Speed was calculated based on the center of mass or a leading foot which simulates a condition with a walkway sensor and validated against manual measurements. Multiple videos of gait over varying distances were obtained by cropping video frames at 0.1-m intervals. Variance in gait speed over specific distances was calculated using ANOVA. “Minimum required distance” was defined as the shortest distance where the confidence interval of gait speed did not exceed the minimal clinically important difference (0.1 m/sec). We also investigated which clinical, anthropometric, or epidemiological variables might influence it by assessing their association with gait speed variance using mean squared error from linear regression.

**Results:**

Gait speed measured by pose estimation (1.55 ± 0.18 m/s) showed a high level of agreement with manual measurement (1.56 ± 0.14 m/s), with an intraclass correlation coefficient of 0.889 (95% CI: 0.822–0.931). “Minimum required distance” was 2.1 m when gait speed was calculated with the center of mass with 95% confidence interval while “minimun required distance” based on a leading foot was 4.7 m with 90% confidence interval. Gait speed itself and muscle strength were positively correlated with gait speed variance (*r* = 0.250, *p* = 0.036 for gait speed; *r* = 0.312, *p* = 0.008 for knee extension strength; *r* = 0.230, *p* = 0.053 for grip strength), whereas other epidemiologic or clinical parameters, and physical performance scales, were not.

**Conclusions:**

Clinically reliable measurement of gait speed could be achieved over 2.1 m using a smartphone and pose estimation inducing the center of mass with 95% confidence interval. The weaker the muscle strength or the slower the gait speed, the shorter the distance might be required.

**Supplementary Information:**

The online version contains supplementary material available at 10.1186/s12877-025-06064-8.

## Introduction

Walking is essential for sustained independence and enhanced quality of life in older adults [1, 2]. It requires coordination and strength, necessitating the integration of the nervous, musculoskeletal, cardiovascular, respiratory, and endocrine systems [2, 3]. Accordingly, gait speed serves as a marker of physiological reserve and overall health status, often referred to as the sixth vital sign [2, 4], and is associated with disability, institutionalization, risk of falls, cognitive decline, mortality, and even life expectancy in older adults [5–8]. It enables the distinction between frail geriatric patients and chronologically aged but functionally preserved older individuals, informs treatment decisions by assessing functional status, and guides strategies to prevent physical disability—a key objective in geriatric medicine and public health [2–4, 6, 9–11].

Despite its importance, the methodology for measuring gait speed varies and primarily follows conventional practices. A recent systematic review indicated considerable variability in testing protocols with little consensus on standardized procedures [12]. Factors such as measurement distance, whether the subject is static or moving at the start point, the presence of a deceleration zone, and floor conditions contribute to inconsistencies [12]. Several guidelines provided recommendations for gait speed measurement [9–11]. For example, the Asian Working Group for Sarcopenia recommends measuring gait speed over a 6-m distance at a normal pace from a moving start without a deceleration phase, and taking the average of two trials for assessment [9]. Similarly, the National Institutes of Health Toolbox protocol measures gait speed over a 4-m course with a static start. This course includes a marked starting line, a 4-m finish line, and a 5-m marker. Participants walk at their usual speed and are instructed to continue walking past the marker [13]. However, implementing this protocol in clinical settings is often challenging due to space constraints. Furthermore, there is no established minimum required distance to ensure clinically meaningful habitual gait speed measurements. Measurements taken over too short a distance may not accurately reflect actual gait speed [14], leading to variability and a lack of standardized procedures.

Advancements in machine learning now allow for gait analysis to be conducted using video recordings with a performance that is comparable to that of traditional motion capture systems [15, 16]. Whereas optical motion capture systems serve as a gold standard for validating other technologies, they require engineering expertise and specialized spaces equipped with multiple cameras [17]. In contrast, pose estimation algorithms can be implemented using a single smartphone, providing quantitative and objective gait analysis with no spatial constraints at a significantly lower cost [16, 18]. Therefore, establishing the minimum required distance for reliable gait speed measurement would maximize the advantages of emerging technologies, allowing gait speed to be widely adopted as the sixth vital sign for patient assessment and treatment planning, even beyond clinical settings. Notably, as the trajectories of the leading foot and center of mass (CoM) differ during gait [19, 20], the minimum required distance may vary depending on the chosen method, such as walkway and pressure sensors or CoM-based approaches, highlighting the need for further investigation.

We hypothesized that the variability of the gait speed measurement would increase monotonically as the measured distance decreased, and gait speed would be clinically meaningful when this variability remained below the minimal clinically important difference (MCID) of the gait speed. Accordingly, this study aimed to determine this minimum required distance using a smartphone camera and pose estimation and to assess whether it is influenced by subject characteristics or measurement methods with a focus on the CoM versus the leading foot.

## Methods

### Participants

Twenty-four healthy, community-dwelling older adults, consisting of 15 men and 9 women with a mean age of 72.1 ± 4.1 years, participated (Table 1). The eligibility criteria of this study were as follows: age ≥ 65 years; ability to walk independently without assistive devices; and absence of conditions that could significantly influence gait, such as neurological disorders (e.g., Alzheimer's disease, Parkinson's disease, or stroke), severe cardiovascular or respiratory impairments with symptoms during daily activities (e.g., heart failure, chronic obstructive pulmonary disease), or musculoskeletal problem that disable independent gait (e.g., joint replacement, spinal surgery, or advanced arthritis).

**Table 1:** Baseline characteristics of study participants

Variables (unit, reference value)	Total (*n* = 24)	Male (*n* = 15)	Female (*n* = 9)	*p*-value
Age (years)	72.1 ± 4.1	73.7 ± 4.2	69.4 ± 1.9	0.009*
Height (m)	1.62 ± 0.07	1.66 ± 0.03	1.53 ± 0.04	< 0.001*
Body weight (kg)	63.7 ± 7.7	66.7 ± 6.1	58.7 ± 7.7	0.010*
BMI (kg/m^2^)	24.4 ± 2.5	24.1 ± 2.04	24.9 ± 3.1	0.429
Gait speed (m/s, < 1.0)^a)^	1.55 ± 0.16	1.57 ± 0.17	1.53 ± 0.16	0.656
Step length (cm)	50.3 ± 6.3	50.9 ± 5.6	49.3 ± 7.7	0.543
Knee extension strength (kgf)	32.4 ± 9.8	35.9 ± 9.6	26.5 ± 7.4	0.019*
Grip strength (kgf, M: < 28, F: < 18)^a)^	27.1 ± 7.7	32.1 ± 5.1	18.8 ± 1.8	< 0.001*
HA-ASM (kg/m^2^, M: < 7.0, F: < 5.7)^a)^	7.14 ± 0.79	7.59 ± 0.49	6.40 ± 0.61	< 0.001*
30 s CST (n, M: ≤ 17, F: ≤ 15)^b)^	16.1 ± 3.6	16.3 ± 3.9	15.8 ± 3.3	0.723
5 T STS (s, ≥ 12)^a)^	9.58 ± 1.47	9.54 ± 1.57	9.66 ± 1.36	0.843
TUG (s, ≥ 12)^b)^	8.11 ± 1.00	8.16 ± 0.93	8.02 ± 1.16	0.755
SPPB (point, ≤ 9)^a)^	11.9 ± 0.3	11.9 ± 0.4	11.9 ± 0.3	0.880
Sarcopenia^a)^ (n)	1	0	1	0.375
Functional sarcopenia^b)^ (n)	5	2	3	0.326

Values are presented as mean ± standard deviation or number

*BMI* body mass index, *HA-ASM* height-adjusted appendicular skeletal muscle mass, *30 s CST* 30-s chair stand test, *5 T STS* 5 times sit-to-stand test, *TUG* timed up-and-go test, *SPPB* short physical performance battery

^a)^Sarcopenia defined according to the Asian Working Group for Sarcopenia guidelines [9]

^b)^Functional Sarcopenia defined according to the Korean Working Group on Sarcopenia guidelines [11]

Reference range from guidelines [9, 11] are provided in parentheses

^*^Statistically significant at *p* < 0.05

### Sample size calculation

The required sample size was determined based on the population within-subject standard deviation (PWSD). The number of subjects was determined to estimate PWSD within 10% of the population value ($\frac{1.96}{\sqrt{2n\left(m-1\right)}}\leq0.1,\;m=the\;number\;of\;observations\;per\;subject)$ using the variance of PSWD ($\frac{{\sigma }_{w}}{\sqrt{2n\left(m-1\right)}}, {\sigma }_{w}=PWSD)$ [21]. A sample size of 24 was required for all of the distances with nine or more observations per subject (m ≥ 9) except for the two longest distances (4.9 and 5-m).

### Muscle mass and strength assessments

Participants underwent bioimpedance analysis using an InBody S10 device (InBody Co., Ltd., Seoul, South Korea) to determine height-adjusted appendicular skeletal muscle mass. Muscle strength was assessed by measuring handgrip and isometric knee extension strength. Handgrip strength was measured using a Takei 5401 Digital Dynamometer (Takei Scientific Instruments Co., Ltd., Niigata, Japan) in a standing position with the elbow fully extended. Isometric knee extension strength was evaluated using a TKK-5710e tension meter (Takei Scientific Instruments Co., Ltd., Niigata, Japan); during measurement, participants were seated on a chair with a dynamometer anchored to it, maintaining knee flexion at 90°. Both measurements were conducted bilaterally, with each side assessed twice and a 1-min rest period between attempts. Participants were instructed to exert maximum effort for each measurement, and the highest reading was used in the analysis. All procedures were conducted by a single trained assessor following the recommendations of Asian Working Group for Sarcopenia and the European Working Group on Sarcopenia in Older People [9, 10].

### Physical performance assessments

Physical performance was evaluated using the Short Physical Performance Battery (SPPB) [22], the 30-s chair stand test [23], the five-times sit-to-stand test [24], and the timed up-and-go test [25]. All assessments were conducted by a single trained assessor in a spacious setting under consistent environmental conditions, following the protocols of the Asian Working Group for Sarcopenia and the European Working Group on Sarcopenia in Older People [9, 10].

### 10-m gait speed test and data acquisition

**Fig. 1:**
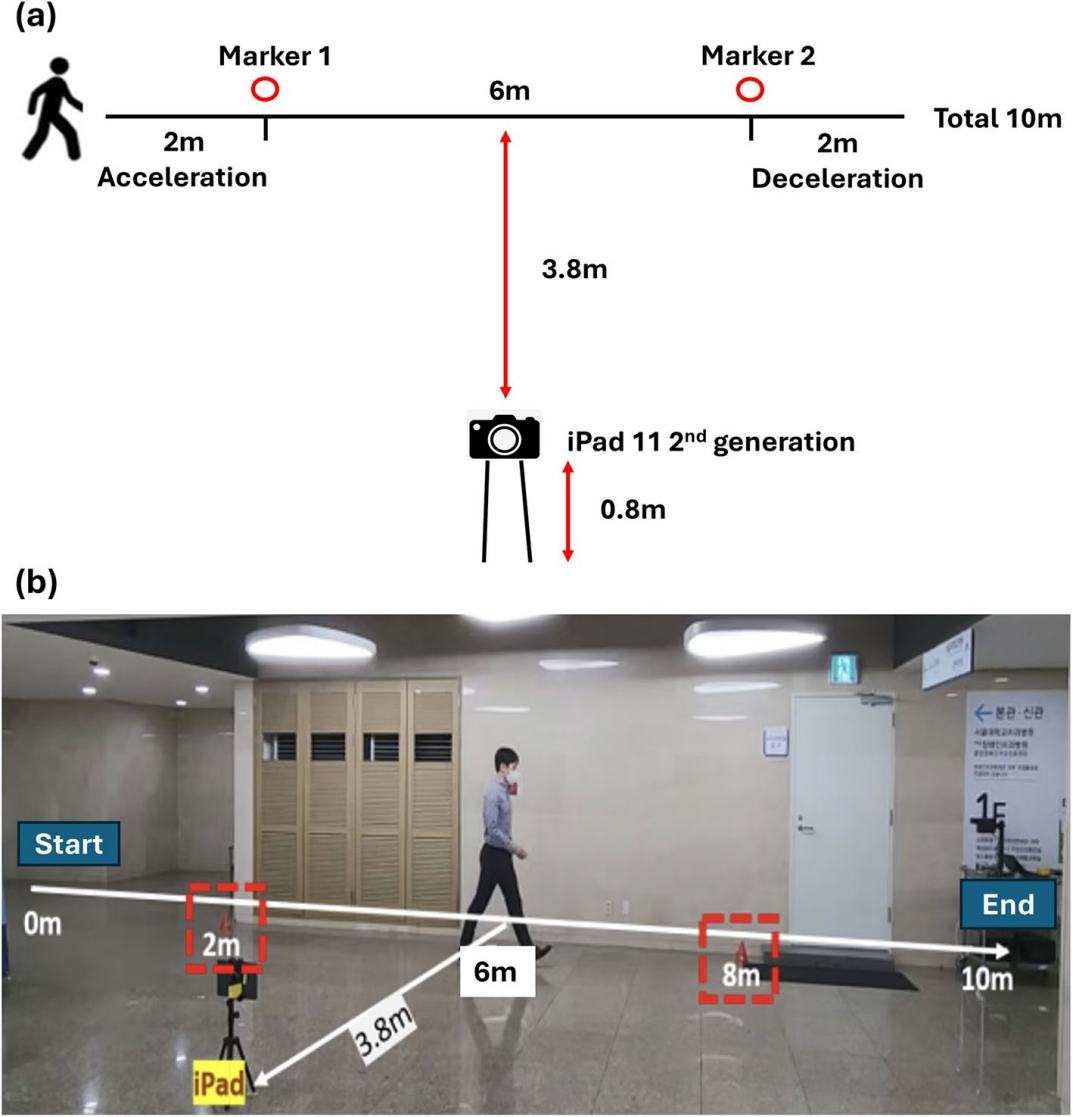
Overview of the experimental set-up. **a** Schematic diagram showing the measurement zones and camera position. **b** Photograph of the setup

Participants walked along a 10-m walkway, which included a 2-m acceleration zone for a dynamic start and a 2-m deceleration zone at the end. They were instructed to walk at their usual pace on a hard surface while wearing comfortable footwear. The 10-m walk was repeated three times, with a minimum rest period of 2 min between trials. Recordings were captured using an Apple iPad Pro 11 2nd Generation (Apple, Inc., Cupertino, CA, USA) equipped with RGB cameras arranged perpendicularly to the walking path at a distance of 3.8 m and a height of 0.8 m. Videos were recorded in the sagittal plane (resolution: 800 × 600 pixels; 30 fps; Fig. 1).

### Gait analysis using 2D pose estimation

**Fig. 2:**
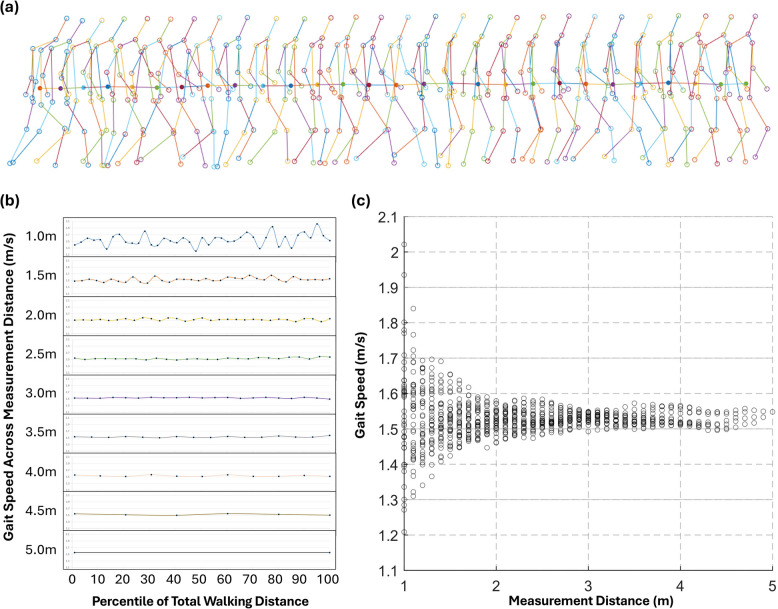
Illustrative case. **a** The movement pattern of the center of mass over time as estimated via pose estimation. **b** Gait speed of each segment according to the measurement distance (1.0–5.0 m). The x-axis represents the percentile of total walking distance (%), and the y-axis represents gait speed (m/s). **c** Distribution of gait speed according to the measurement distance

A customized pose estimation model (ViFive, Inc., Boulder, CO, USA) was used, which tracked 14 key body points using an architecture adapted from a standard stacked hourglass model [26]. We introduced multiple objectives to enhance the context, accuracy, speed, and stability of the model, which are vital for musculoskeletal assessment. The classification model included a random forest classifier with optimized features to increase accuracy and speed while reducing the model size. Pixel-per-meter estimation used markers at 2 and 8 m (Fig. 1). The CoM of each subject was determined using the weighted sums of the body segment centers of mass (Fig. 2a).

### Gait speed estimation

Gait speed was measured using two independent methods for validation: manually with a stopwatch and using pose estimation algorithms. Manually assessed speed was determined by an evaluator using a stopwatch to record the time taken for the subjects to pass by the markers set at 2 and 8 m. Pose estimation gait speed was calculated by dividing the distance covered between frames by the elapsed time using either the CoM or the leading foot as reference points. CoM-referenced measurements simulate those obtained via conventional motion capture system, whereas leading foot-referenced measurements simulate those made using walkway or pressure sensors such as GAITRite® (CIR Systems Inc., Franklin, NJ, USA).

### Gait speed measurement validation

Gait speeds measured using a stopwatch and pose estimation were compared using a linear mixed-effects model, with speed over 6 (manual) or 5 m (pose estimation) per trial as the dependent variable and with subject random effect to account for multiple tests from each subject. The intraclass correlation coefficient (ICC) was used to evaluate absolute agreement between gait speed measurements obtained via the two methods for the same walking trials.

### Change of uncertainty with measured distance

A 5-m walk video of a skeleton with 14 key points was extracted from each recording using our pose estimation algorithm. This was further edited by cropping at 0.1-m intervals to generate 4.9- to 1.0-m segments. One 5.0-m walk video generated two 4.9-m segments, three 4.8-m segments, and so forth, up to 41 segments for a 1.0-m walk, culminating in 861 segments of varying distance (Fig. 2b,c).

The variability of gait speed across the measured distances was defined as the within-subject standard deviation (WSD) for each measured distance, calculated as the square root of the mean-square error in a one-way analysis of variance, where groups combined subjects with distance intervals. Three gait speed data from three measurements were collected for each group to avoid underestimating within-subject variation due to overlapping distances when distance intervals are not considered. For example, for a 4.7-m walk, four gait speed measurements were obtained at 4.7-m distances (0–4.7, 0.1–4.8, 0.2–4.9, and 0.3–5.0 m), and within-subject variation at a 4.7-m distance was estimated by considering different distance intervals.

### Determination of minimum required distance

To determine the minimum required distance, we utilized WSD at each measured distance. Given that confidence intervals (CI) quantify variability, we computed the half-width of the CI using WSD and the critical value corresponding to the chosen confidence level. Specifically, the 95% CI was calculated as 1.96 × WSD, and the 90% CI as 1.64 × WSD. For the measurement to be clinically meaningful, the half-width of the CI, reflecting gait speed variability, had to remain below the MCID of 0.1 m/s [27, 28]. Thus, the minimum required distance was defined as the shortest distance at which this criterion was met, ensuring that gait speed measurements remained within an acceptable range of variability.

### Factors affecting gait speed variability

CoM trajectory was plotted as distance against time for each test. Linear regression analysis provided a trend line and the mean squared error (MSE) for each subject. As MSE quantifies deviations from the trend line, lower MSE values indicate less variability in gait speed, leading to a shorter minimum required distance. We investigated whether epidemiological, anthropometric, or clinical variables were associated with MSE using linear regression following Pearson’s correlation for continuous variables and point-biserial correlation for dichotomous variables to identify subject characteristics influencing the minimum required distance. All processing and statistical analyses were conducted using MATLAB R2023b (MathWorks, Natick, MA, USA) and SAS 9.4 (SAS Institute, Cary, NC, USA), with statistical significance set at *p* < 0.05.

## Results

### Validation of gait speed measurement using pose estimation

The linear mixed-effects model to compare the mean gait speed values obtained via manual assessment and the pose estimation algorithm revealed no statistically significant difference (*p* = 0.794). Additionally, the intraclass correlation coefficient between the two methods demonstrated a high level of agreement (ICC = 0.889), with 95% confidence intervals ranging from 0.822 to 0.931, as shown in Table 2.

**Table 2:** Reliability of gait speed measurement in different methods

	Manual assessment	Pose estimation	ICC (95% CI)
Gait speed (m/s)	1.56 ± 0.14	1.55 ± 0.18	0.889 (0.822 to 0.931)

Gait speed values are expressed as mean ± standard deviation

*ICC* Intraclass correlation coefficient, *CI* Confidence interval

### Determination of minimum required distance for clinically reliable gait speed measurement

**Fig. 3:**
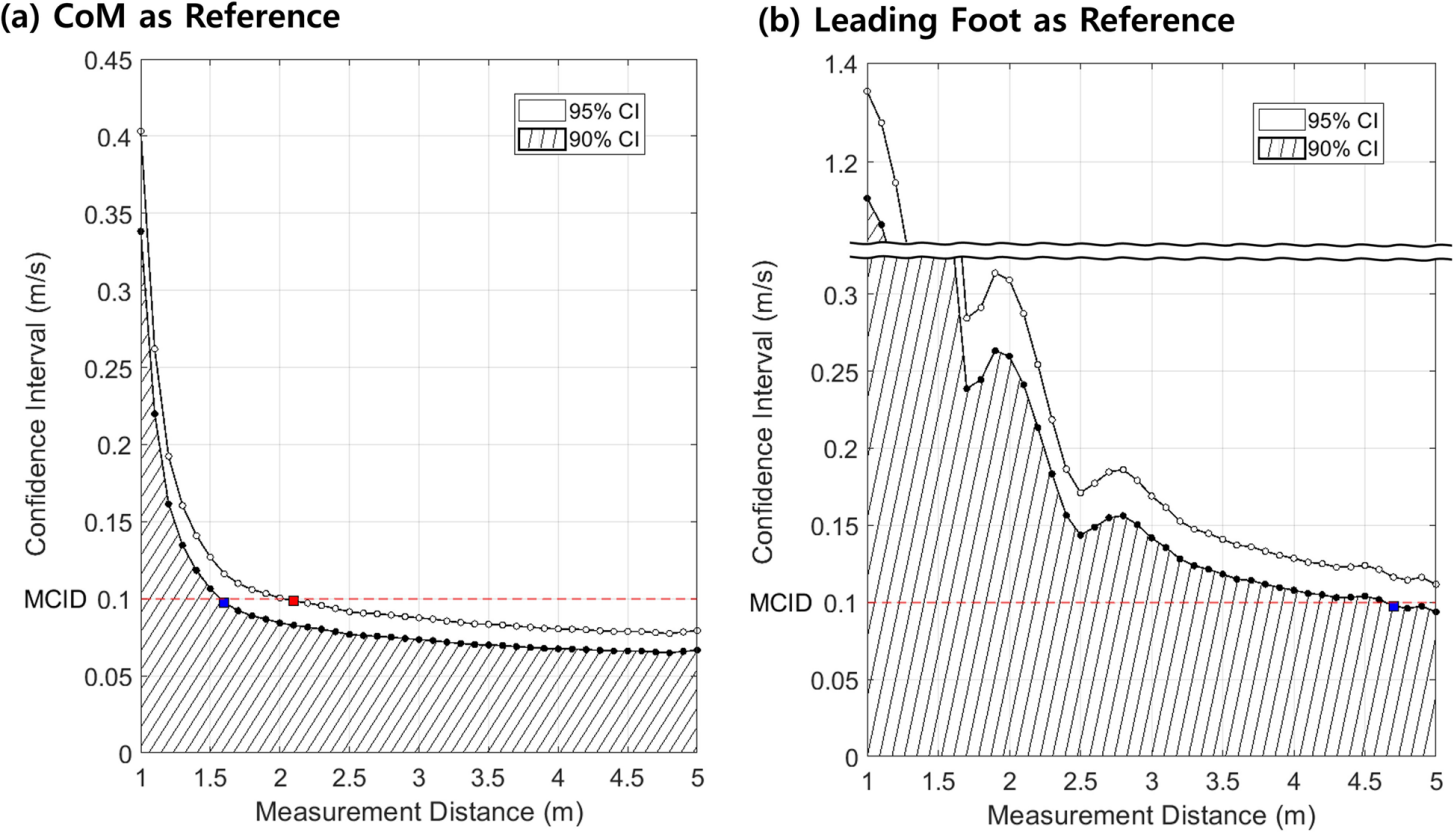
Relationship between measurement distance and confidence interval of gait speed (standard deviation × Z-value). Dashed lines indicate where the CI boundaries correspond to the MCID of 0.1 m/s. Red square: minimum required distance for 95% CI (CoM: 2.1 m). Blue square: minimum required distance for 90% CI (CoM: 1.6 m, leading foot: 4.7 m). **a** Uses CoM as the reference, while (**b**) uses the leading foot as the reference. MCID: minimal clinically important difference, CoM: center of mass

To achieve reliable gait speed measurements, a minimum distance of 2.1 m was required when using the CoM as the reference point, as indicated by the 95% CI. When a confidence level of 90% was applied, the minimum required distance decreased to 1.6 m. In contrast, using the leading foot as the reference did not yield reliable measurements at distances under 5 m, based on a 95% CI; when a confidence level of 90% was applied, the minimum required distance was achieved at 4.7 m (Supplementary Table 1, Fig. 3).

### Factors influencing the minimum required distance for gait measurement

**Fig. 4:**
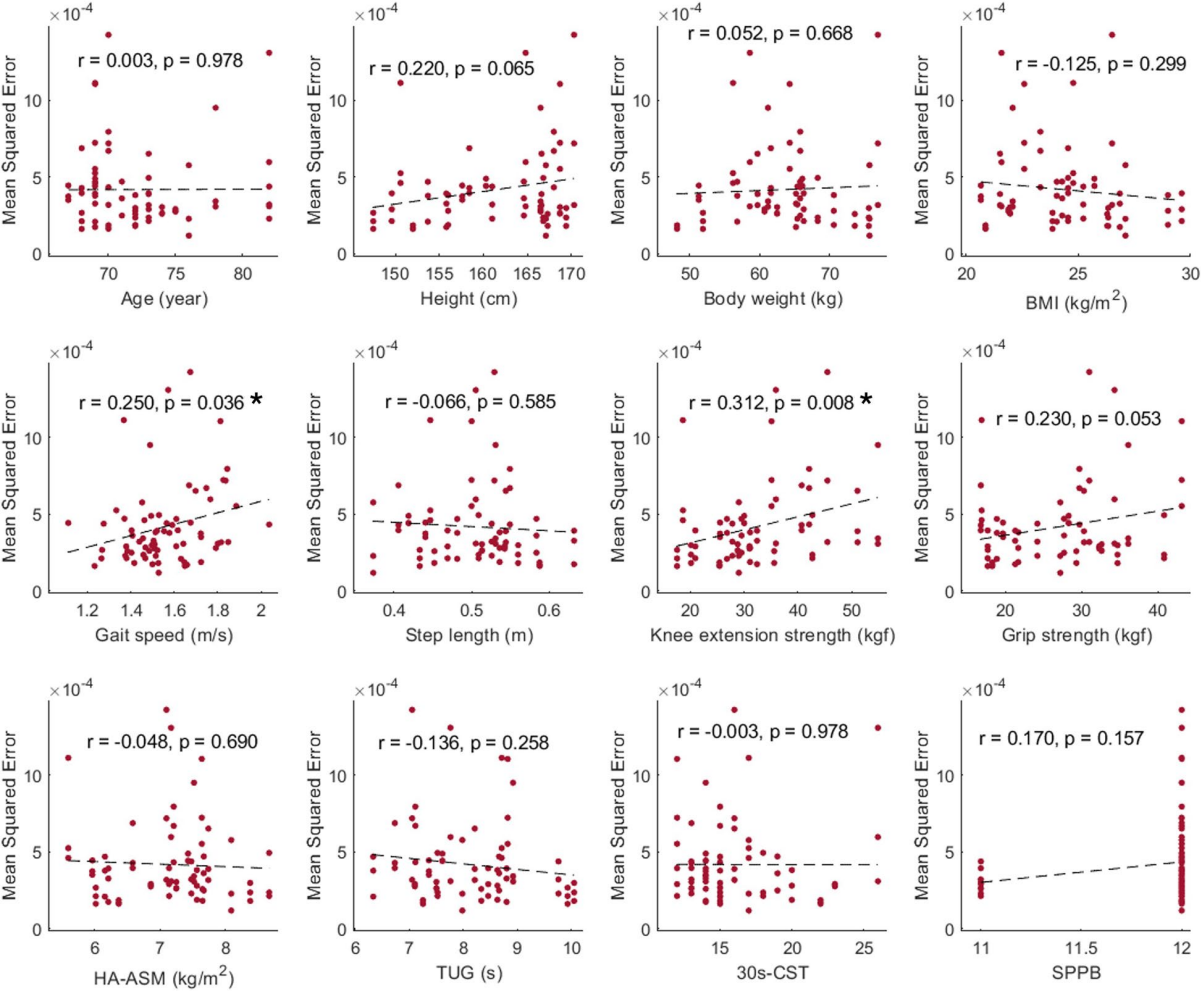
Correlation between mean squared error and variables related to subject characteristics. BMI: body mass index, HA-ASM: height-adjusted appendicular skeletal muscle mass, TUG: timed up-and-go test, 30 s CST: 30-s chair stand test, SPPB: short physical performance battery. *Statistically significant at *p* < 0.05

Variability in gait speed within an individual session was significantly associated with gait speed (*r* = 0.250; *p* = 0.036) and knee extension strength (*r* = 0.312; *p* = 0.008). While correlations with height (*r* = 0.220; *p* = 0.065) and grip strength (*r* = 0.230; *p* = 0.053) did not reach statistical significance (*p* < 0.05), they tended towards positive correlation (*p* < 0.10). No significant correlations were found with other variables, including age, body weight, body mass index, step length, height-adjusted appendicular skeletal muscle mass, chair stand test, timed up-and-go test, SPPB, or sex, which showed no significant association even after adjusting for other variables (Fig. 4).

## Discussion

### Assessment of minimum distance requirements for clinically reliable gait measurement using pose estimation

This study aimed to determine the minimum required distance for the clinically reliable measurement of habitual gait speed in healthy older individuals using a pose estimation algorithm and RGB camera, compare different body reference points, and identify the clinical factors influencing this distance. The findings indicate that CoM-referenced measurements require a minimum distance of 2.1 m, whereas leading foot-referenced measurements necessitate longer distances. Additionally, individuals with higher gait speed and greater knee extension strength exhibit greater gait variability, necessitating a longer minimum distance for clinically reliable gait speed measurement.

One significant advantage of utilizing 2D RGB cameras and pose estimation over traditional methods such as manual measurement and motion capture systems is the technical liberation from spatiotemporal constraints. This engineering advancement has prompted a preliminary consensus on the minimum distance for clinically acceptable gait speed. Several previous studies have investigated the reliability of gait speed measurements based on measurement distance. Kim et al. [29] compared the reliability of gait speed according to distance (4, 6, and 10 m) among community-dwelling older individuals, finding that reliability increased with distance. Similarly, Fernández-Huerta et al. [30] evaluated gait speed measurements at 4 and 10 m and reported a slight improvement in consistency over a longer distance. Another systematic review suggested that a distance ranging from 2.4 to 3.0 m was likely sufficient for measuring usual gait speed with conventional manual methods, based on a study that compared two gait speed measurements over 8 (2.4 m) and 20 feet (6.1 m); a difference of 0.01 m/s was found, which was statistically but not clinically significant [12, 31]. However, the differences in speed between distances cannot account for speed uncertainty at a specific distance, and no direct evidence has been suggested for the minimum required distance while focusing on changes in measurement variability. In this study, we determined the minimum distance at which the confidence interval did not exceed the MCID for gait speed measurements. The MCID was set at 0.1 m/s based on previous studies involving older adults [27, 28]; a change in gait speed of 0.1 m/s has been associated with increased survival among older adults, underscoring the clinical relevance of this threshold [6].

### Interpretation of the minimum required distance

In this study, we proposed a minimum required distance for clinically reliable gait speed of 2.1 m, corresponding to approximately two strides (four steps) in our test subjects. These results can be interpreted in two ways. The measured length should not be reduced to a single gait cycle, no matter what body part is used as a reference point. Although the CoM showed the most stable trajectory, it also oscillates vertically and horizontally during the gait cycle [20, 32, 33]. The horizontal velocity along the x-axis fluctuated beyond the limit of the MCID within the gait cycle; because of this, if the measured segment is shorter than the stride length, variability increases exponentially and cannot be overcome by technical advancements (Fig. 3). Additionally, two stride lengths after 2 m of acceleration were sufficient to provide a clinically reliable gait speed. This signifies that the stride-to-stride variation is remarkably small in healthy older individuals, though this may depend on the walking environment and subject characteristics [34, 35]. Because of this, we analyzed whether gait variability was affected by epidemiologic, anthropometric, and clinical parameters.

### Influencing factors

Gait variability within a fixed distance of 5 m was positively correlated with overall gait speed and knee extension strength; positive but insignificant trends were also observed for grip strength and height. These findings align with those of Tesio et al. [36], who documented a U-shaped relationship between the anterior and posterior oscillation of the CoM and gait speed, indicating a complex interaction between biomechanical movements and speed. The overall path length of the CoM and the anterior–posterior oscillation were smallest around a gait speed of 1.1 m/s, where the metabolic cost of walking was minimized; as the speed increased beyond this point, oscillation gradually increased [36]. The average gait speed of the participants in this study was 1.55 ± 0.16 m/s, suggesting that the increase in CoM oscillation with increased gait speed was reflected in the increased fluctuation of gait speed measurement. Furthermore, the observed positive correlation between knee extension strength and gait speed variability may be attributed to the stronger association between knee extension strength and gait speed itself (*r* = 0.485, *p* = 0.016). Additionally, gait speed, muscle strength, and height may affect the stride length. Given that approximately two strides are required for clinically reliable gait speed measurement, an increase in stride length may lead to a corresponding increase in the minimum required distance. However, physical performance tests, such as the timed-up-and-go test and the 30-s chair stand test, were not identified as influencing factors. This lack of association may be attributed to the multifactorial nature of these assessments, which reflect complex pathophysiological mechanisms [9].

This study also explored whether measurement methods affected the minimum required distance. Using the leading foot as a reference point was intended to simulate systems utilizing walkway sensors, such as infrared sensors, or pressure sensors. The results indicated that these systems required a longer distance than CoM-based methods for clinically reliable gait speed measurement. The horizontal velocity of the foot fluctuates to a greater range throughout the gait cycle than that of the CoM [19, 37]. Although the possibility that pose estimation algorithms were more susceptible to determining the positions of extremities than those of proximal body parts was not rejected, the CoM still oscillates less during gait than any other body part. Because of this, measuring gait speed in reference to the CoM if available can be recommended to acquire a more reliable gait speed.

### Limitations and future directions

Despite these insights, this study was subject to several limitations worth noting. First, the small sample size and unequal distribution of sex may limit the generalizability of the findings. Moreover, homogeneous participant demographics were included, which may not represent variability in gait among various populations. Neurological conditions such as mild cognitive impairment or Parkinson’s disease as well as orthopedic conditions such as spinal stenosis or advanced degenerative arthritis are common in older patients, whose gait function may offer different features from those of other populations. Furthermore, this study did not account for participants’ physical activity levels, which are associated with physical performance. Future studies should explore the minimum required distance in a broader demographic and clinical spectrum, incorporating assessments of physical activity levels and including a larger sample size to enhance external validity. In addition, pose estimation algorithms and portable smart devices are rapidly developing. We attempted to adopt a commercially available state-of-the-art technology for the current study; however, more advanced hardware and software may report different minimum required distances for gait speed tests.

In conclusion, reliable gait speed measurements using 2D RGB cameras and pose estimation are achievable among healthy older adults at a minimum distance of 2.1 m. This method not only requires shorter distances than traditional approaches but also enhances reliability, supporting its broader application in clinical and research settings. When assessing gait speed in older adults with suspected functional decline, clinicians should consider that individuals with higher gait speed and greater muscle strength may exhibit increased gait variability, necessitating a longer minimum required distance for reliable measurement. Further research is warranted to validate these findings in larger, more diverse populations and settings and to enhance the clinical applicability of gait assessments.

## Supplementary Information

Supplementary Material 1.

## Data Availability

The datasets used and analysed during the current study are available from the corresponding author on reasonable request.

## Abbreviations

CoM: Center of mass
MCID: Minimal clinically important difference
PWSD: Population within-subject standard deviation
SPPB: Short Physical Performance Batter
WSD: Within-subject standard deviation
CI: Confidence interval
MSE: Mean squared error
