# Supplementary material for: Minimum required distance for clinically significant measurement of habitual gait speed
Source: BMC Geriatr. 2025 Jul 5;25:497. doi: 10.1186/s12877-025-06064-8 (PMC12229044; doi:10.1186/s12877-025-06064-8)
Supplement: Supplementary file 1 — Supplementary Material 1. [file 12877_2025_6064_MOESM1_ESM.docx]

# **SUPPLEMENTARY MATERIALS**

**Minimum Required Distance for Clinically Significant Measurement of Habitual Gait Speed**

**Supplementary Table 1**. Standard deviation of gait speed according to measurement distance using CoM and leading foot as reference points

| Distance (m) | SD (CoM) | SD (Leading foot) |
| --- | --- | --- |
| 5.0 | 0.04047 | 0.05698 |
| 4.9 | 0.04001 | 0.05935 |
| 4.8 | 0.03948 | 0.05842 |
| 4.7 | 0.03978 | 0.05938 |
| 4.6 | 0.04015 | 0.06188 |
| 4.5 | 0.04023 | 0.06324 |
| 4.4 | 0.04018 | 0.06276 |
| 4.3 | 0.04039 | 0.06267 |
| 4.2 | 0.04073 | 0.06381 |
| 4.1 | 0.04093 | 0.06429 |
| 4.0 | 0.04103 | 0.06561 |
| 3.9 | 0.04123 | 0.06660 |
| 3.8 | 0.04157 | 0.06794 |
| 3.7 | 0.04190 | 0.06941 |
| 3.6 | 0.04234 | 0.06996 |
| 3.5 | 0.04255 | 0.07191 |
| 3.4 | 0.04272 | 0.07385 |
| 3.3 | 0.04315 | 0.07522 |
| 3.2 | 0.04363 | 0.07788 |
| 3.1 | 0.04430 | 0.08245 |
| 3.0 | 0.04467 | 0.08621 |
| 2.9 | 0.04510 | 0.09138 |
| 2.8 | 0.04559 | 0.09495 |
| 2.7 | 0.04600 | 0.09418 |
| 2.6 | 0.04623 | 0.09047 |
| 2.5 | 0.04670 | 0.08730 |
| 2.4 | 0.04776 | 0.09510 |
| 2.3 | 0.04876 | 0.11146 |
| 2.2 | 0.04956 | 0.12973 |
| 2.1 | 0.05029 | 0.14663 |
| 2.0 | 0.05125 | 0.15779 |
| 1.9 | 0.05273 | 0.16003 |
| 1.8 | 0.05403 | 0.14860 |
| 1.7 | 0.05610 | 0.14504 |
| 1.6 | 0.05930 | 0.20370 |
| 1.5 | 0.06475 | 0.30651 |
| 1.4 | 0.07193 | 0.41354 |
| 1.3 | 0.08187 | 0.50937 |
| 1.2 | 0.0982 | 0.59089 |
| 1.1 | 0.13365 | 0.65267 |
| 1.0 | 0.20569 | 0.68499 |

CoM: center of mass, SD: standard deviation.
